# Nucleophosmin leukemogenic mutant activates Wnt signaling during zebrafish development

**DOI:** 10.18632/oncotarget.10878

**Published:** 2016-07-28

**Authors:** Elisa Barbieri, Gianluca Deflorian, Federica Pezzimenti, Debora Valli, Marco Saia, Natalia Meani, Alicja M. Gruszka, Myriam Alcalay

**Affiliations:** ^1^ Department of Experimental Oncology, Istituto Europeo di Oncologia, Milan, Italy; ^2^ The FIRC Institute of Molecular Oncology (IFOM) Foundation, Milan, Italy; ^3^ Dipartimento di Oncologia ed Emato-Oncologia, Università degli Studi di Milano, Milan, Italy; ^4^ Current address: Gene Expression and Regulation Program, The Wistar Institute, Philadelphia, PA, USA

**Keywords:** acute myeloid leukemia, nucleophosmin, zebrafish, primitive hematopoiesis, Wnt signaling

## Abstract

Nucleophosmin (NPM1) is a ubiquitous multifunctional phosphoprotein with both oncogenic and tumor suppressor functions. Mutations of the *NPM1* gene are the most frequent genetic alterations in acute myeloid leukemia (AML) and result in the expression of a mutant protein with aberrant cytoplasmic localization, NPMc+. Although NPMc+ causes myeloproliferation and AML in animal models, its mechanism of action remains largely unknown. Here we report that NPMc+ activates canonical Wnt signaling during the early phases of zebrafish development and determines a Wnt-dependent increase in the number of progenitor cells during primitive hematopoiesis. Coherently, the canonical Wnt pathway is active in AML blasts bearing NPMc+ and depletion of the mutant protein in the patient derived OCI-AML3 cell line leads to a decrease in the levels of active β-catenin and of Wnt target genes. Our results reveal a novel function of NPMc+ and provide insight into the molecular pathogenesis of AML bearing *NPM1* mutations.

## INTRODUCTION

Nucleophosmin (NPM1) is a ubiquitous nucleolar phosphoprotein that shuttles continuously between the nucleus and the cytoplasm [1, 2]. It is involved in a wide range of cellular functions, including ribosome biogenesis and transport, centrosome duplication, and cell cycle regulation (reviewed in [3]). NPM1 binds to a variety of cellular proteins, including p53 [4], Arf [5] and c-Myc [6] and controls their localization and stability.

Structural or functional abnormalities of NPM1 are present in both solid tumors and hematological malignancies. Of particular relevance, about one third of acute myeloid leukemia (AML) cases are associated with mutations in the C-terminal region of the protein, which result in the loss of a nucleolar localization signal and the gain of a *de novo* nuclear export signal [7, 8]. Consequently, the mutant protein (NPMc+) is delocalized to the cytoplasm [8]. NPMc+ AML was recognized as a separate provisional entity in the 2008 revision of the World Health Organization classification of myeloid neoplasms and acute leukemia [9] since it possesses distinct features that include a specific gene expression profile, the association with a normal karyotype, the involvement of different hematopoietic lineages, and clinically, a favorable prognosis.

Different animal models have been generated to investigate the causal relationship between NPMc+ expression and the development of AML [10]. Knockdown of endogenous *npm1a* in zebrafish embryos resulted in a reduction in the number of myeloid cells that was rescuable by human NPM1. Conversely, the expression of NPMc+ led to an expansion of primitive and definitive hematopoietic cells [11]. Ablation of the *Npm1* gene in mice triggered early embryonic lethality, whereas animals expressing *NPMc+* in the hematopoietic system developed a myeloproliferative disease [12] and leukemia [13, 14]. In particular, activation of a humanized NPMc+ knock-in allele in the mouse hematopoietic system caused *Hox* gene overexpression, a feature shared with NPMc+ AML patients [15], and enhanced self-renewal and myelopoiesis. Importantly, one third of the animals developed late-onset AML, suggesting that NPMc+ alone is insufficient for leukemic transformation and requires additional cooperating mutations [13]. In line with this hypothesis, mice carrying both the humanized *NPMc+* allele and a knock-in allele coding for the FLT3-ITD mutation develop AML with short latency and full penetrance [16].

A common feature in AML is constitutive activation of the Wnt pathway, which plays a pivotal role in HSC maintenance and in the differentiation of blood cells [17, 18]. Canonical Wnt signalling is triggered by the interaction of a soluble Wnt ligand with a member of the Frizzled family of receptors, which results in stabilization and activation of β-catenin. Activated β-catenin enters the nucleus and binds to transcriptional co-activators of the TCF/LEF1 family inducing expression of target genes. In the absence of Wnt ligands, β-catenin is sequestered in a multiprotein cytoplasmic complex (“destruction” complex) and degraded in the proteasome [19, 20]. Inhibition of the canonical pathway can be achieved by expression of Dickkopf (Dkk) proteins, a group of secreted molecules that bind to Wnt receptors preventing their activation [21]. Signaling through Wnt pathway is required for different aspects of early embryonic development [22], including morphogenetic movements and cell type specification, and its deregulation leads to mislocalization of future adult tissues.

We found that the leukemogenic NPMc+ mutant activates canonical Wnt signaling during zebrafish development causing an expansion of the hematopoietic progenitor pool in primitive zebrafish hematopoiesis. Wnt signaling was indeed responsible for the myeloproliferative phenotype, since it was rescued by the overexpression of the *dkk1b* Wnt inhibitor. In addition, we established that canonical Wnt signaling is active in the patient derived OCI-AML3 cell line that expresses NPMc+ and in AML blasts expressing NPMc+.

Our study provides new insight into the molecular mechanisms underlying NPMc+ function, suggesting the involvement of Wnt activation in the establishment and/or the progression of NPMc+ AML.

## RESULTS

### NPMc+ expression in zebrafish alters the morphology of developing embryos

**Figure 1:**
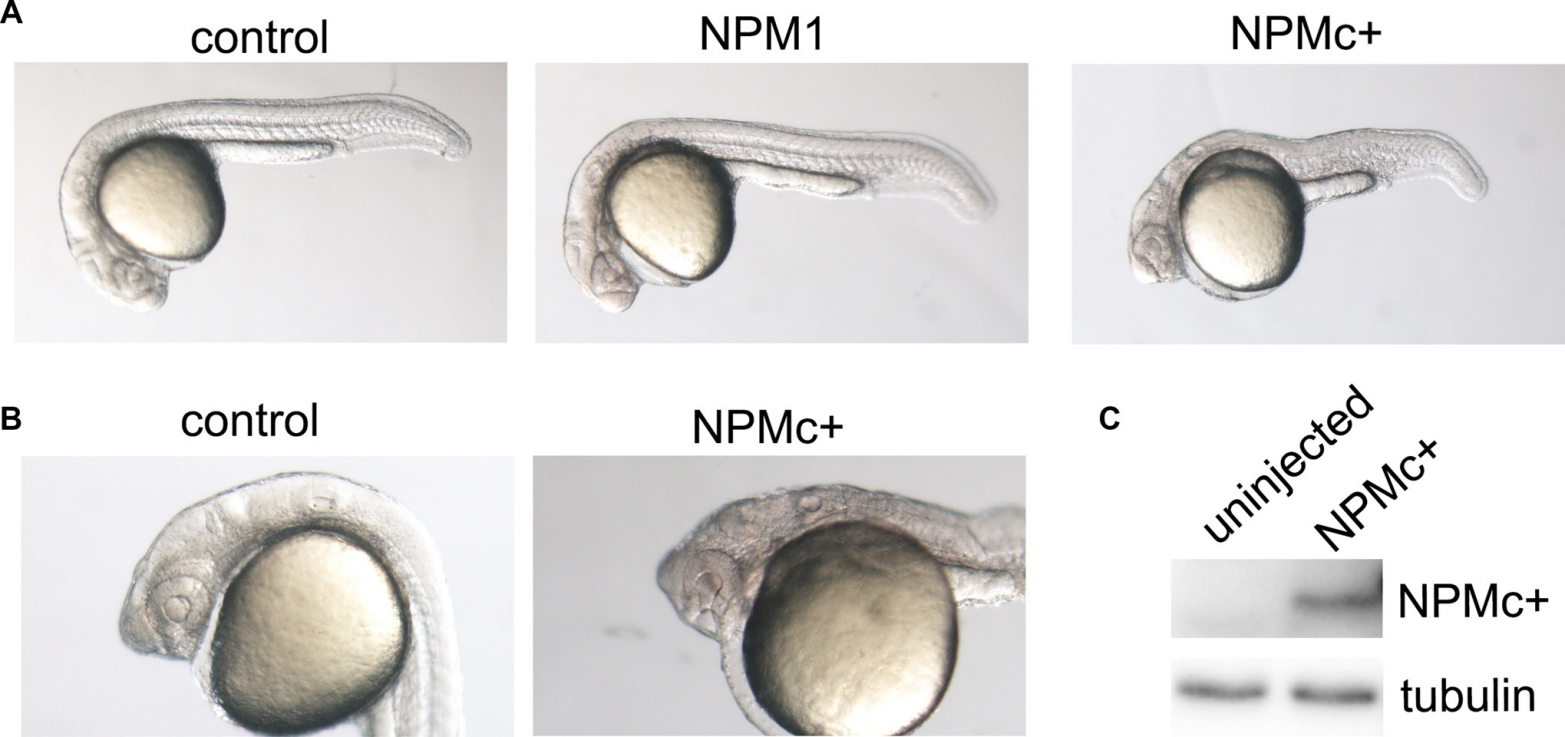
Embryo morphology after NPM1 and NPMc+ expression in zebrafish embryos (**A**) Overall view of control (uninjected), NPM1 (90% of injected embryos showing the reported phenotype) and NPMc+ (85% of injected embryos showing the reported phenotype in multiple experiments) expressing embryos at 24 hpf. (**B**) Head structures in control and NPMc+ expressing embryos at 24 hpf. (**C**) Western blot analysis of NPMc+ expression levels in zebrafish embryos.

One-cell stage zebrafish embryos were injected with 100 pg of *in vitro* transcribed mRNA representative of the most common *NPM1* mutation (mutation A) or with 120 pg of wild-type (wt) human *NPM1*. The morphology of the injected embryos was analyzed at 24 hours post fertilization (hpf). *NPMc+* expression led to a general decrease in the body size and an enlargement of the hematopoietic region (Figure 1A), whereas expression of the wt *NPM1* had a minimal or no effect (Figure 1A). Interestingly, the most affected embryos showed a strong posteriorization of the head, lacking the anterior part of the telencephalon, and presented defects in eye development (Figure 1B). Expression of NPMc+ protein in zebrafish embryos was confirmed by western blot (Figure 1C).

The phenotype observed upon NPMc+ expression resembled that of embryos bearing mutations that lead to the activation of Wnt signaling (*wnt8*, *axin1*), as reported in literature [23, 24], suggesting that injection of *NPMc+* mRNA may result in an enhanced activity of the Wnt pathway.

### NPMc+ modulates CE movements during zebrafish gastrulation

**Figure 2:**
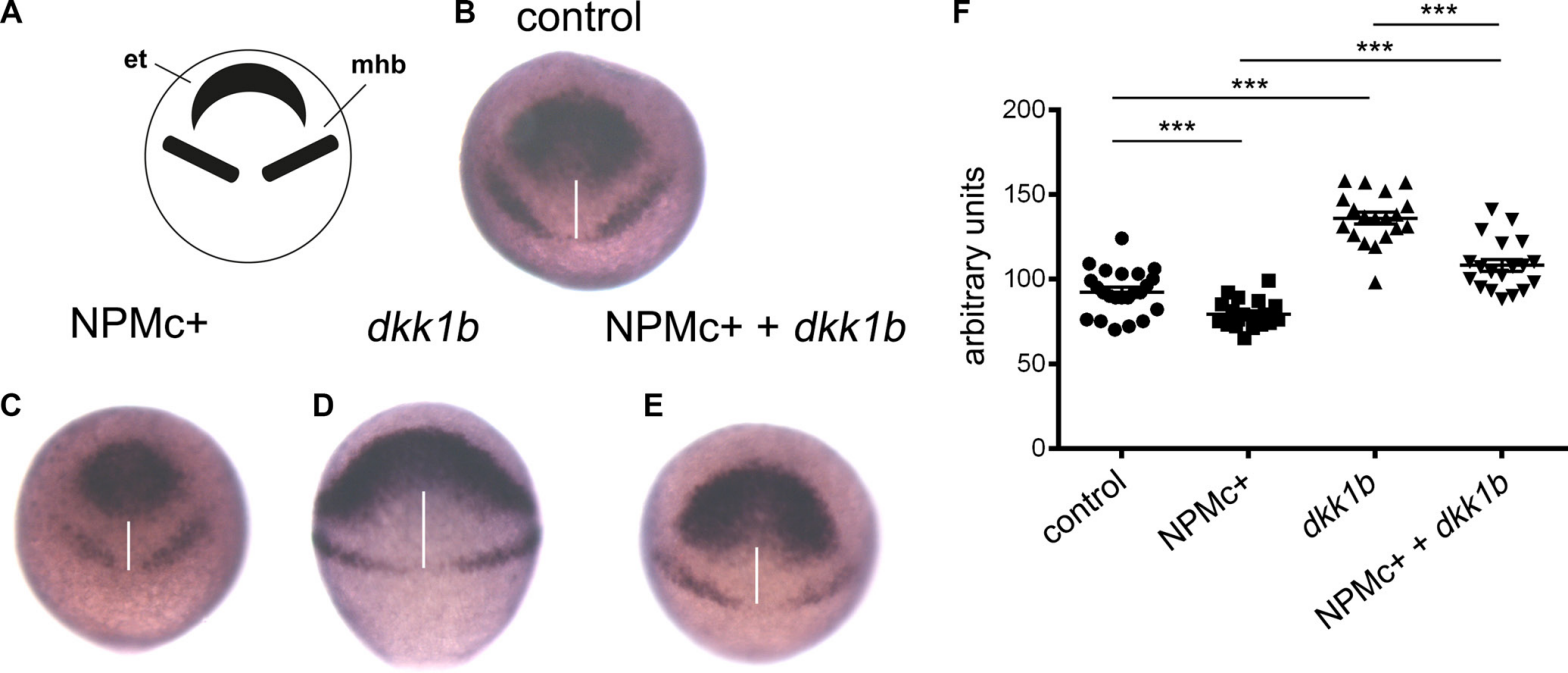
Analysis of CE movements at gastrulation Whole mount *in situ* hybridization for *rx3* and *pax2a* markers of the eye field and telencephalon region (et) and mid-hindbrain boundary (mhb), respectively. All embryos are at 90% epiboly, dorsal view. Distance between *rx3* and *pax2a* expression regions was measured with ImageJ; statistical significance was assessed with a Student't test. (**A**) Schematic representation of areas of markers expression. (**B**) Representative example of uninjected control. Embryos injected with: (**C**) *NPMc*+ mRNA; (**D**) *dkk1b* mRNA; (**E**) *NPMc*+ and *dkk1b* mRNAs. (**F**) Graphic representation of the distance between *rx3* and *pax2a* expression regions for each measured sample (20 embryos were analyzed for each condition).

The posteriorized phenotype observed upon NPMc+ expression could be caused by a block of convergence and extension (CE) movements during gastrulation, when the correct positioning of presumptive tissues is tightly regulated. In particular, the distance between the regions that will give rise to the eye/telencephalon and the mid-hindbrain boundary is controlled by canonical Wnt signaling [25]. We studied the consequences of NPMc+ expression on these CE movements by whole mount *in situ* hybridization experiments using a combination of probes that allow the identification of the two neural structures – the eye/telencephalon region characterized by the expression of *rx3* and the mid-hindbrain border cells expressing *pax2a* at 90% epiboly (9 hpf) (Figure 2A, 2B). The expression of NPMc+ reduced the distance between the regions highlighted by the two probes, indicating a reduction of the interval between the eye/telencephalon and the mid-hindbrain border (Figure 2C). Conversely, the injection of 50 pg of zebrafish *dkk1b* mRNA, an inhibitor of the canonical Wnt pathway [21], led to a strong increase in the reciprocal distance between the two regions (Figure 2D). Co-injection of the two mRNAs partially rescued their respective effects (Figure 2E). The differences observed were statistically significant (Figure 2F). These results suggest that NPMc+ has the capacity to activate the canonical Wnt pathway during development of the anterior central nervous system in zebrafish, resulting in the deregulation of CE movements during gastrulation.

### NPMc+ activates canonical Wnt signaling in zebrafish embryos

**Figure 3:**
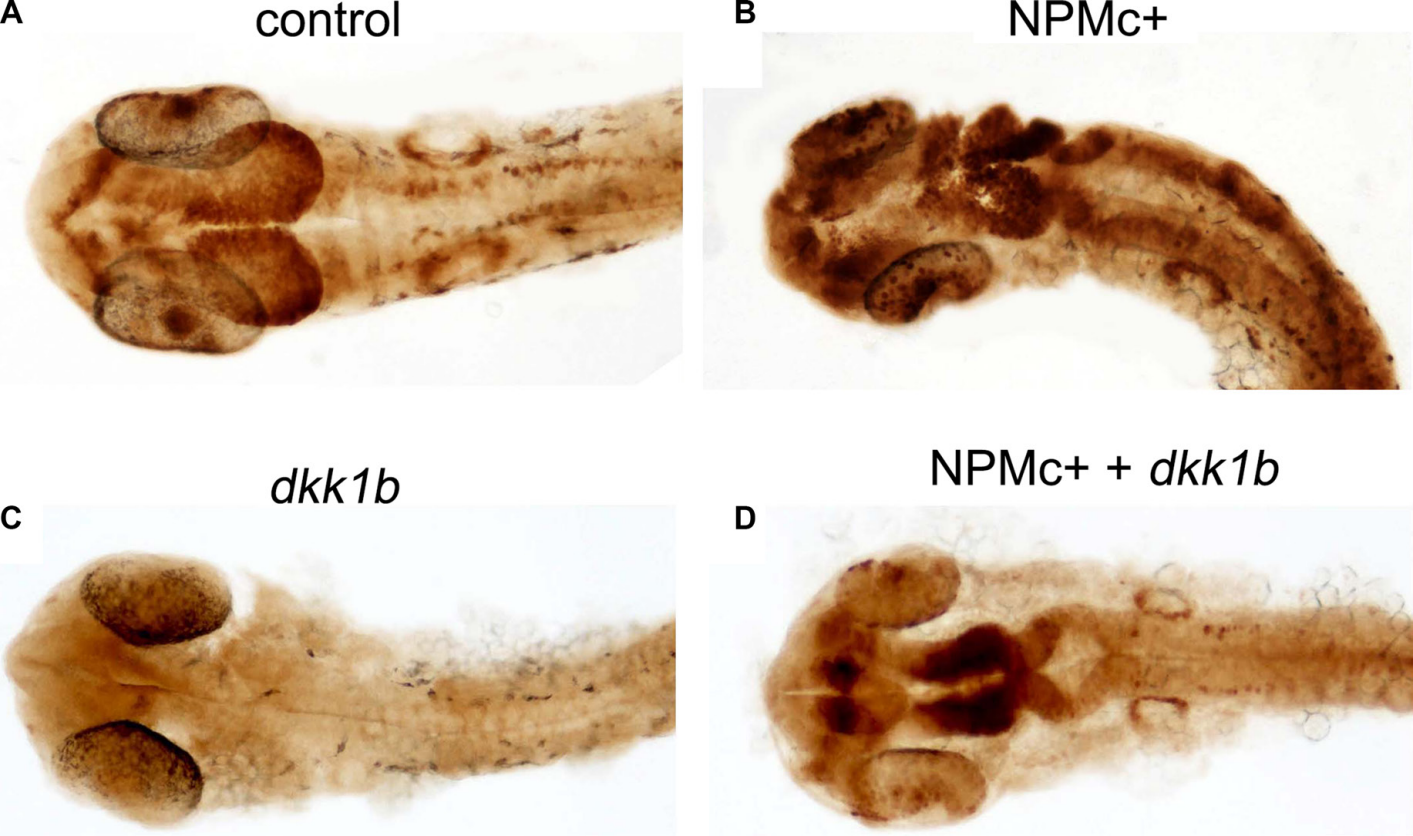
Canonical Wnt activation in 28 hpf embryos DAB-staining for GFP expression in *TOP:GFP* embryos at 28 hpf. Embryos are shown in dorsal view. (**A**) uninjected control (32/32). Embryos injected with: (**B**) *NPMc*+ mRNA (27/28); (**C**) *dkk1b* mRNA (32/32); (**D**) *NPMc*+ and *dkk1b* mRNAs (16/22).

We next investigated if activation of Wnt signaling by NPMc+ is maintained in zebrafish embryos at later stages of development. The *Tg(TOP:GFP)* transgenic line, which bears the GFP reporter gene controlled by four enhancers and the basal promoter of *lef1*, a β-catenin-dependent transcription factor [26], was used for this purpose. *Tg(TOP:GFP)* embryos were injected with *NPMc+* mRNA or *dkk1b* mRNA. Due to the low level of fluorescence in transgenic embryos, GFP expression was revealed at 28 hpf using an anti-GFP antibody and DAB staining. Expression of *NPMc+* increased the β-catenin signal (Figure 3A, 3B) whereas *dkk1b* strongly decreased the GFP signal (Figure 3C). Co-injection of *dkk1b* and *NPMc+* mRNAs rescued the signal (Figure 3D), suggesting that activation of canonical Wnt signaling by NPMc+ is maintained at later stages of zebrafish development.

### The increase of hematopoietic progenitors after NPMc+ expression is Wnt-dependent

Since the NPMc+ mutant is specific of AML, we next studied the hematopoietic compartment during development. In zebrafish two distinct waves of hematopoiesis occur, i.e. primitive hematopoiesis, characteristic of embryonic stages, and definitive hematopoiesis that gives rise to blood cells in the adult animal. During primitive hematopoiesis, common precursors of hematopoietic and endothelial cells (hemangioblasts) appear at two different sites: the anterior lateral mesoderm (ALM) and the posterior lateral mesoderm (PLM). Later on during development, the two stripes of the PLM converge and give rise to the intermediate cell mass (ICM) while a transient wave of hematopoiesis occurs in the posterior blood island (PBI) [27]. In the ICM, progenitors give rise predominantly to erythrocytes, whereas in the PBI only a transient population of myeloid cells emerges. At 28–30 hpf, primitive mature leukocytes can be observed respectively in the ICM and PBI regions and throughout the body, while differentiated erythrocytes are dispersed in the embryo blood.

**Figure 4:**
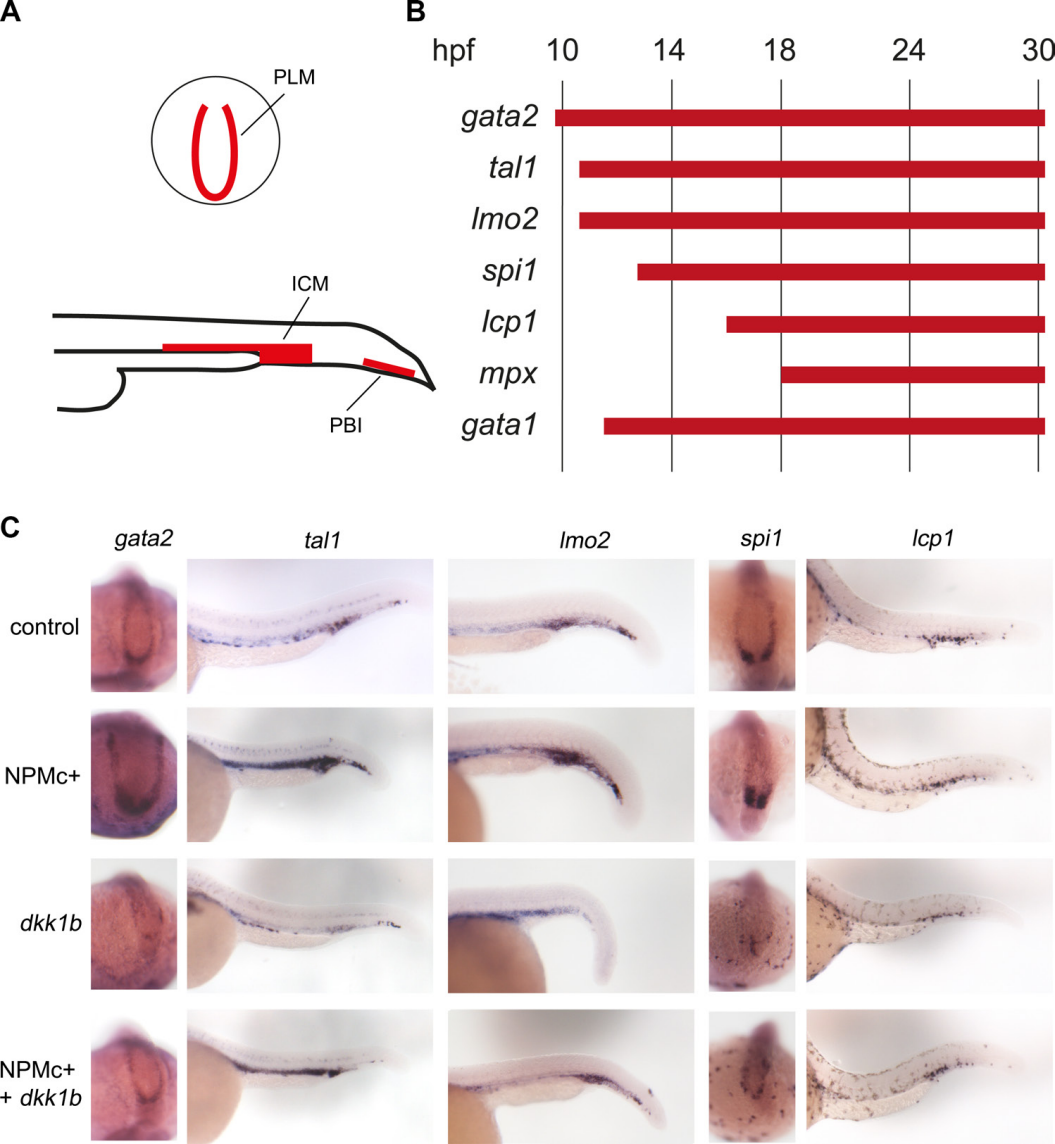
Expression of hematopoietic markers during early zebrafish hematopoiesis (**A**) Schematic representation of primitive hematopoietic organs in zebrafish embryos as seen in posterior (top) and lateral (bottom) views showing PLM (posterior lateral mesoderm), ICM (intermediate cell mass) and PBI (posterior blood island). (**B**) Timeline of expression of hematopoietic markers expressed in the posterior hematopoietic region of zebrafish embryos during primitive hematopoiesis. (**C**) Whole mount *in situ* hybridization for *gata2* (15 somites embryos, dorsal and posterior view. The phenotypes were encountered in a number of embryos as detailed for each marker hereafter. Control: 41/41, *NPMc+*: 29/32, *dkk1b*: 31/31, *NPMc+* and *dkk1b*: 20/27), *tal1* and *lmo2* (24 hpf embryos, lateral view. For *tal1*: control: 51/52, *NPMc+*: 65/85, *dkk1b*: 30/43, *NPMc+* and *dkk1b*: 17/34. For *lmo2*: control: 44/45, *NPMc+*: 33/34, *dkk1b*: 27/33, *NPMc+* and *dkk1b*: 24/33), *spi1* (15 somites embryos, dorsal and posterior view. Control: 32/32, *NPMc+*: 21/23, *dkk1b*: 33/34, *NPMc+* and *dkk1b*: 22/28), *lcp1*(30 hpf embryos, lateral view. Control: 23/24, *NPMc+*: 26/28, *dkk1b*: 25/25, *NPMc+* and *dkk1b*: 30/37).

Whole mount *in situ* hybridization experiments were performed with the aim of analyzing the expression of known hematopoietic markers in zebrafish embryos expressing NPMc+. Since anterior structures may be deformed or absent in severely posteriorized embryos, therefore skewing the evaluation of hematopoietic cell numbers in the ALM, the analysis was restricted to the posterior structures (Figure 4A). Hematopoietic markers were analyzed at different time points according to their expression pattern: g*ata2, tal1* and *lmo2* were evaluated in early hematopoietic progenitors, *spi1 (pu.1)* in myeloid progenitors, *gata1* in cells of the erythroid lineage and *lcp1* and *mpx* (*mpo*) in differentiated myeloid cells [27]. A detailed scheme of the markers used in this study and a timeline of their expression during zebrafish development is shown in Figure 4B.

The injection of *NPMc*+ into one-cell stage embryos led to an increase in the expression of *gata2* at 16 hpf, and of *tal1* and *lmo2* at 24 hpf, revealing an expansion of the pool of primitive progenitors in the PLM and in the ICM (Figure 4C). The hybridization signals were, instead, faint for all three markers in *dkk1b* overexpressing embryos (Figure 4C). Co-injection of *dkk1b* and *NPMc*+ mRNA rescued the phenotype in all cases (Figure 4C), suggesting that the effect elicited by *NPMc*+ depends on the activation of Wnt signaling.

Next, primitive myeloid precursors, characterized by expression of the pu.1 transcription factor [27] transcribed from the *spi1* gene, were studied at 16 hpf. As expected, the pool of *spi1* positive cells increased in *NPMc+* injected embryos. Conversely, the number of these cells decreased in *dkk1b* overexpressing embryos (Figure 4C). Moreover, the injection of *dkk1b* mRNA led to the dispersion of the few *spi1* positive cells in the posterior region of the embryos and the co-injection of *NPMc+* with *dkk1b* only partially rescued the phenotype (Figure 4C). It appears, therefore, that the previously reported increase in the pool of myeloid precursors upon NPMc+ expression [11], as defined by *spi1* positivity at early stages of development, is partially reversible by Wnt signaling inhibition.

To assess if the increase of progenitor cells observed at 16 hpf and 24 hpf corresponds to the expansion of mature blood cells, we performed *in situ* hybridization experiments using markers of differentiated cells. In *NPMc+* injected embryos, the pool of leukocytes (*lcp1+*) was similar to uninjected controls and was also not modified by the injection of *dkk1b* mRNA or the combination of the two mRNAs (Figure 4C). A similar pattern was observed when analyzing the granulocytes in *mpo*:GFP transgenic embryos (data not shown). The expansion of the pool of hematopoietic progenitors was, therefore, not strictly connected to an increase in the number of more differentiated cells.

The effect of NPMc+ on erythropoiesis was investigated in *gata1*:dsRED embryos. *Gata1* expression levels were not modified by *NPMc+* mRNA expression at 24 hpf (Supplementary Figure S1), suggesting that the erythroid lineage is not altered by the presence of the leukemogenic mutant. On the contrary, the injection of *dkk1b* mRNA had a strong negative effect, with a considerable decrease of the *gata1+* cells (Supplementary Figure S1). The co-injection of the two mRNAs enhanced the expression of *gata1* compared to *dkk1b* alone, but positive cells were more concentrated in the primitive hematopoietic region (Supplementary Figure S1), confirming that NPMc+ partially rescues the effect of overexpression of *dkk1b* in zebrafish embryos.

In conclusion, these experiments showed that NPMc+ expression led to an expansion of the pool of progenitor cells during primitive zebrafish haematopoiesis and this effect was rescued by overexpression of the Wnt inhibitor *dkk1b*.

### NPMc+ silencing in AML-OCI3 cells leads to a decrease in active β-catenin and AXIN2 expression levels

Activation of Wnt signaling has been reported in AML blasts expressing leukemogenic fusion proteins [17, 28, 29] but has not been described, to date, in NPMc+ AML. On the basis of our results in zebrafish, we investigated Wnt signaling activation in the OCI-AML3 cell line, which was derived from a patient with NPMc+ AML.

**Figure 5:**
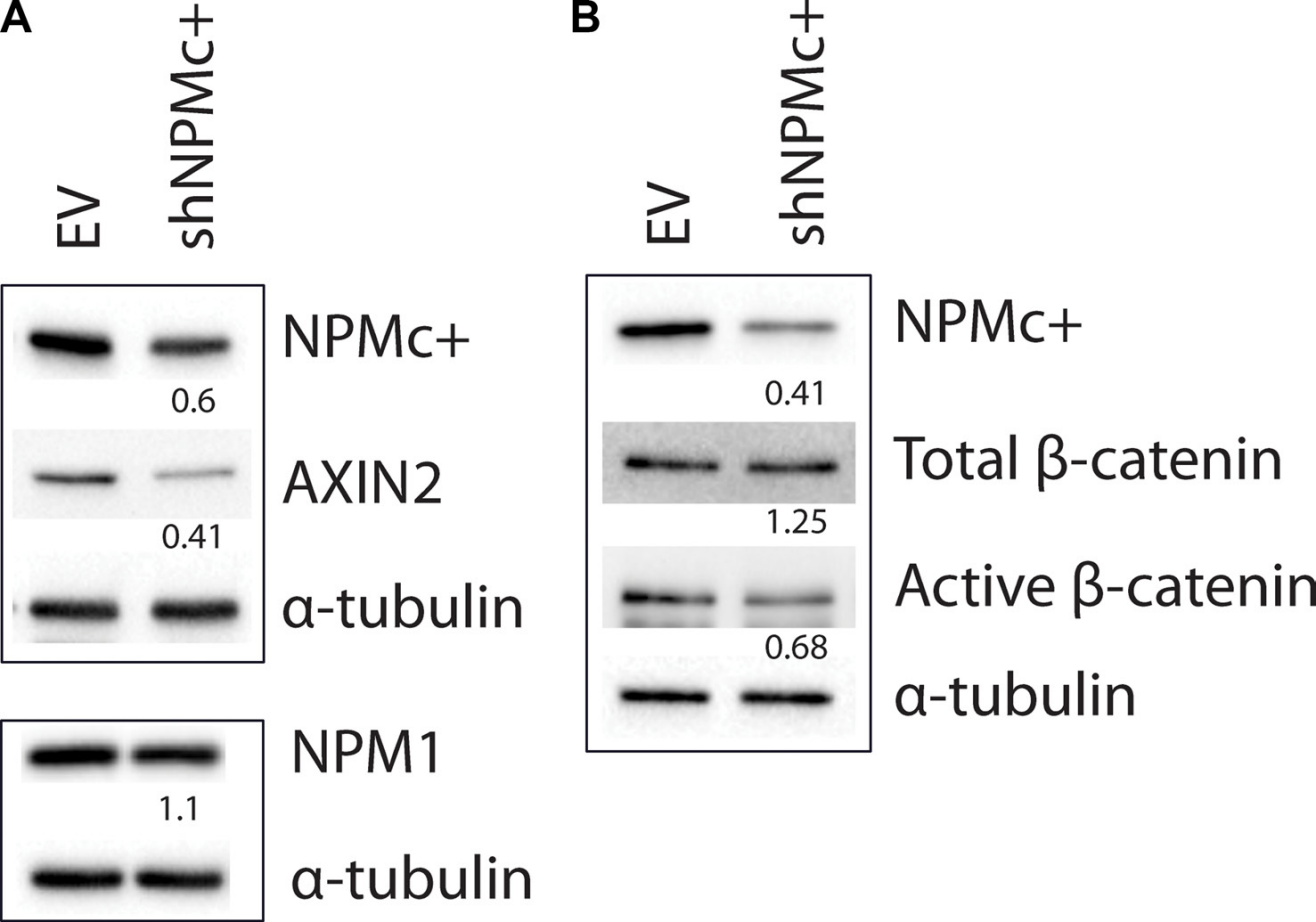
Expression of AXIN2 and active β-catenin in OCI-AML3 cells after silencing NPMc+ (**A**) Transduction of shNPMc+ in OCI-AML3 cells results in approximately 50% reduction of NPMc+ protein levels (top panel), but does not affect NPM1 expression (bottom panel). Expression of AXIN2 decreases in shNPMc+ treated OCI-AML3 cells. (**B**) Total β-catenin levels remained unaltered after NPMc+ silencing whereas active β-catenin expression decreases proportionally to NPMc+. Western blots in A and B were generated using lysates from different transduction experiments. The top and bottom panels in A correspond to different gels produced with the same lysates. Numbers below the lanes correspond to the relative quantity of each protein in shNPMc+ treated cells compared to controls, normalized using α-tubulin expression. Images were analyzed and quantified with ChemiDoc™ MP system software (BioRad).

A significant reduction of NPMc+ expression was achieved after transduction of OCI-AML3 cells with a short-hairpin RNA that specifically targets the *NPM1* mutant (Figure 5A top panel and 5B). The levels of wild type NPM1 protein were not modified by this treatment (Figure 5A bottom panel). Expression of total β-catenin was slightly increased after NPMc+ depletion, whereas the levels of active β-catenin (Figure 5B) and of the Wnt targets AXIN2 (Figure 5A) and CyclinD1 (not shown) decreased significantly. This result suggests that NPMc+ expression correlates with the activation of canonical Wnt signaling in OCI-AML3 cells.

### Blasts from patients with NPMc+ AML show activation of the canonical Wnt pathway

**Figure 6:**
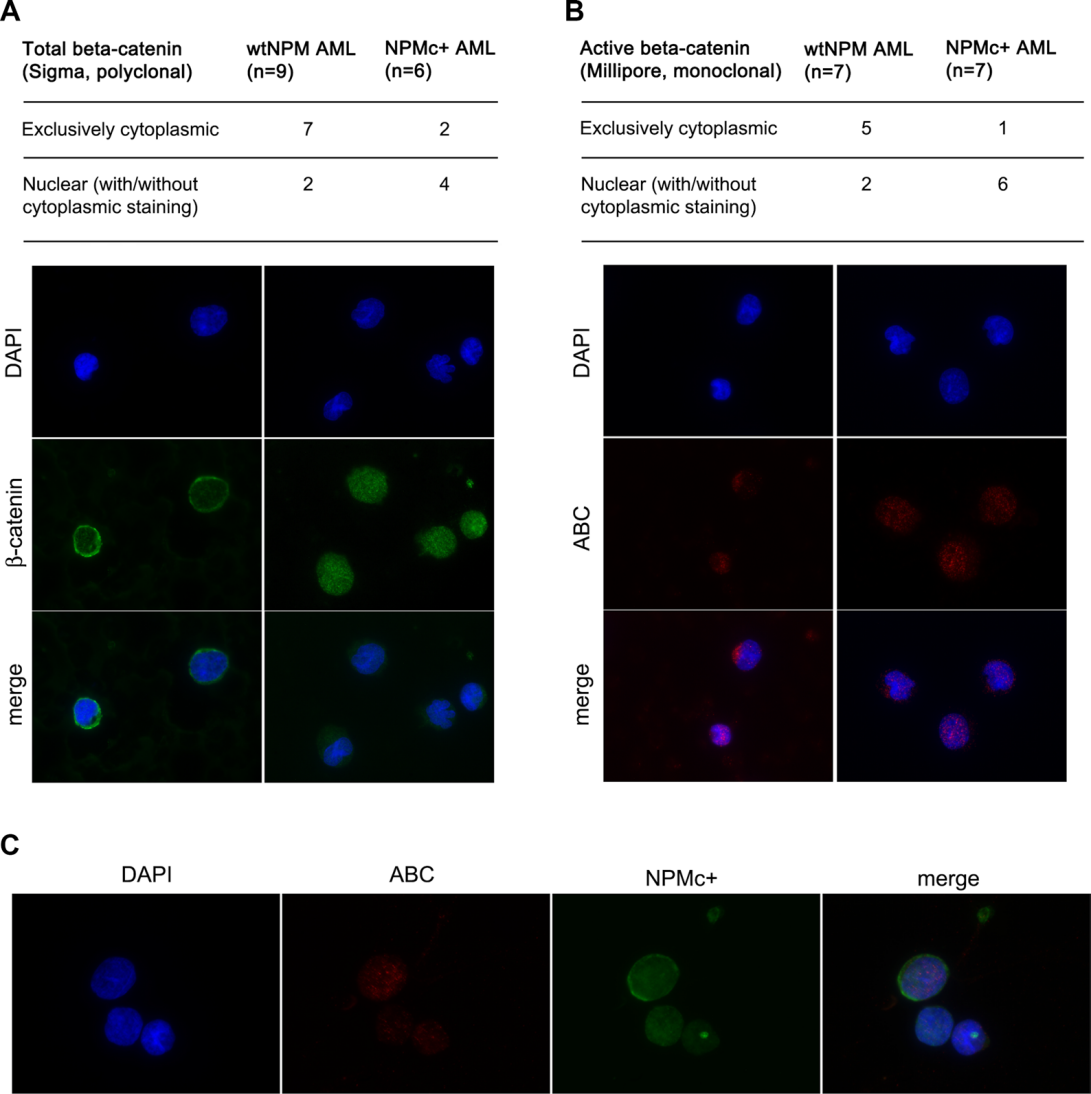
Analysis of β-catenin localization in AML patients (**A**) Summary of results obtained with an antibody that recognizes total β-catenin (Sigma) and examples of staining patterns: cytoplasmic (left panels) and mostly nuclear (right panels). (**B**) Summary of results obtained with an antibody that specifically recognizes active β-catenin (ABC) (Millipore) and examples of staining patterns: cytoplasmic (left panels) and mostly nuclear (right panels). (**C**) Example of a co-staining performed on a BM smear of an NPMc+ AML patient showing strong nuclear ABC signal in a cell that expresses high levels of NPMc+. Staining with anti β-catenin, anti-ABC or anti-NPMc+, DAPI staining and merged channels (Merge) are shown at magnification of x600.

Next, we analyzed by immunofluorescence the subcellular localization of β-catenin on bone marrow smears from a series of AML patients. First, a polyclonal antibody that recognizes all forms of β-catenin was used. Four out of six patients (67%) bearing *NPM1* mutations and two out of nine patients (22%) with wild-type *NPM1* displayed a variable proportion of β-catenin in the nucleus (Figure 6A). An antibody that specifically recognizes the active form of β-catenin (anti-ABC) revealed nuclear staining in six out of seven patients (87%) with *NPM1* mutations and two out of seven patients (29%) with wild-type *NPM1* (Figure 6B). In some cases, anti-ABC disclosed a staining pattern restricted to the nucleus, such as that shown in the right hand panels of Figure 6A and 6B representing cells from the same patient stained with the two antibodies. Finally, we used the anti-ABC antibody in conjunction with an antibody against NPMc+ (Figure 6C) and found that cells with high levels of NPMc+ displayed a nuclear localization of β-catenin.

**Figure 7:**
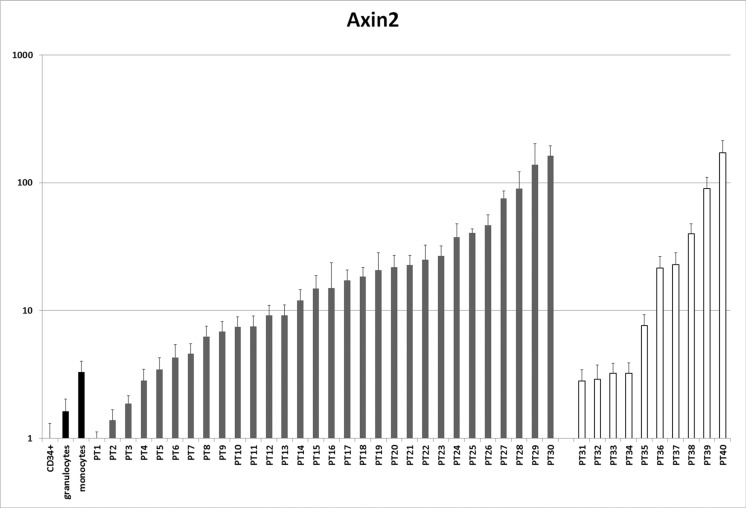
Analysis of AXIN2 expression in AML patients mRNA levels of the Wnt target *AXIN2* were assessed by qPCR in a series of patients with AML with normal karyotype versus normal controls (CD34+ cells, granulocytes and monocytes). Patients 1–30 have NPMc+ AML, patients 31–40 do not have NPM mutations. See Supplementary Table S1 for other patient characteristics.

mRNA levels of the Wnt target gene *AXIN2* [30] were analyzed by qPCR in blasts from 40 AML patients with normal karyotype (30 with NPM mutations and 10 without) and in normal hematopoietic cells (CD34+ progenitors, monocytes and granulocytes, Figure 7). Compared to CD34+ precursors, *AXIN2* levels were higher in more differentiated cells (1.6-fold in granulocytes and 3.3-fold in monocytes, respectively). In 27/30 NPMc+ AML patients (90%) *AXIN2* levels were increased by >2-fold when compared to CD34+ cells. 26/30 cases (87%) displayed increased *AXIN2* levels compared to granulocytes, and 22/30 (73%) compared to monocytes. AML with normal karyotype without *NPM1* mutations also displayed increased expression of *AXIN2* in 10/10 cases (100%) when compared to CD34+ cells, 7/10 cases (70%) compared to granulocytes, and 6/10 (60%) compared to monocytes. There was no correlation between *AXIN2* levels and concurrent mutations in other genes that are frequently mutated in AML such as *FLT3, IDH1, IDH2, DNMT3a, N-RAS* and *K-RAS* (Supplementary Table S1).

It appears, therefore, that canonical Wnt signaling is activated in most cases of AML with normal karyotype. The mechanism of Wnt activation in AML without NPM mutations remains to be identified.

## DISCUSSION

A myeloproliferative effect of NPMc+ has been described both in zebrafish [11] and mouse [12]. Our study shows that, in zebrafish, this effect derives from activation of Wnt signaling by NPMc+, since co-expression of *dkk1b* rescued the phenotype. Interestingly, NPMc+ only affects precursors without expanding the pool of mature myeloid cells in primitive hematopoiesis. This discrepancy can be explained by the dose-dependent sensitivity of different hematopoietic cells to Wnt activation [18]. In fact, HSC are stimulated to proliferate with mild levels of Wnt activation, while in the presence of a potent activation the HSC pool is depleted and myeloid differentiation is enhanced [18]. Activation of the Wnt pathway by NPMc+ may be sufficient to trigger proliferation of progenitor cells but insufficient to elicit an effect on the differentiation of myeloid cells.

Canonical Wnt signaling is known to be active in HSC [31] and in AML expressing specific oncogenes such as MLL-AF9, AML1-ETO and PML-RARα [17, 28, 29]. We found that Wnt signaling is also active in blasts derived from patients bearing *NPM1* mutations as indicated by the nuclear accumulation of active β-catenin and overexpression of one of its target genes, *AXIN2*. This finding suggests that NPMc+ may be responsible for Wnt signaling activation in an AML subtype without known translocation products. Interestingly, it has been previously suggested that leukemic stem cells strictly depend on the integrity and activation of the Wnt pathway whereas adult HSC do not require β-catenin for self-renewal [29]. Clearly, such difference may offer an opportunity for new therapeutic approaches in AML including the NPMc+ AML subtype.

Zebrafish embryos expressing NPMc+ showed a morphology that suggests an impairment in the formation of the anteroposterior axis, a function that involves modulation of Wnt signaling. In the definition of anteroposterior identity, an important role is also played by *Hox* genes and their upstream modulators, including the caudal-related (*Cdx*) genes. Interestingly, zebrafish mutants for *Cdx* genes have a bloodless phenotype, revealing their importance in directing mesodermal cells toward the hematopoietic fate [32], and canonical Wnt signaling has been shown to activate the *Cdx/Hox* axis [33]. The characteristic increase in *Hox* gene expression found in NPMc+ AML [15] may, therefore, partly derive from activation of canonical Wnt signaling.

In conclusion, we describe a novel function of NPMc+, the leukemogenic mutant of nucleophosmin, namely, activation of the canonical Wnt pathway leading to an expansion of the pool of primitive hematopoietic progenitors in a Wnt-dependent manner.

## MATERIALS AND METHODS

### Strains and maintenance

Tübingen wild-type and transgenic Tg(*gata1*:dsRED) [34], Tg(*mpx*:GFP) [35], Tg(*TOP*:GFP) [26] zebrafish strains were maintained and bred according to standard procedures [36].

### RNA injection

Zebrafish *dkk1b*, human *NPM1* and *NPMc+* cDNAs were cloned into *pCS2+* plasmid. 10 μg of each construct were digested with *NotI*, purified and *in vitro* transcribed using the *mMessage mMachine SP6 kit* (Ambion) to generate capped RNAs. Zebrafish embryos were microinjected in the yolk or in the cell at 1-cell stage using a combination of the following: 120 pg synthetic human *NPM1* mRNA, 100 pg of *NPMc+* mRNA and 50 pg of *dkk1b* mRNA.

### Whole-mount *in situ* hybridization and immunostaining

The RNA probes for *in situ* hybridization were synthesized as follows: 1 μg of each vector was linearized with the corresponding restriction enzyme and *in vitro* transcribed with T7, T3 or SP6 RNA polymerase (for specific restriction condition see Supplementary Table S2). Next, RNA probes were labeled with digoxigenin (DIG) using DIG-RNA labeling mix (Roche). Embryos were fixed overnight at 4°C with 4% paraformaldehyde in PBS solution. Whole mount *in situ* hybridization was performed as previously described [37].

In *Tg(TOP:GFP)* transgenic embryos, GFP expression was revealed with an antibody against GFP (TP401, Torrey Pines Biolabs) and subsequently by colorimetric reaction using peroxidase, conjugated to the secondary antibody, and DAB (3,3′-Diaminobenzidine). Embryos were then included in 85% glycerol, flat-mounted and viewed with a Nikon stereomicroscope.

### Western blot

Zebrafish embryos at 24 hpf were lysed in SDS buffer, a mixture of 1 part of buffer I (150 mM Tris-HCl pH6.8, 30% glycerol and 5%SDS) and 3 parts of buffer II (25 mM Tris-HCl pH 8.3, 50 mM NaCl, 0.5% NP-40, 0.5% sodium deoxycholate, 0.1% SDS) with proteases inhibitors. 50 μg of each sample were loaded on a SDS-PAGE gel and presence of NPMc+ was detected using T26 antibody [38]. Anti-human tubulin (Sigma-Aldrich) was used as loading control.

### Interference anti-NPMc+ in OCI-AML3 cell line

OCI-AML3 cells, which express NPMc+ mutant protein (mutant A) were transduced with pSicoPuroR lentiviral vector expressing a short hairpin anti-NPMc+-mutant A (target sequence: 5′-gatctctgtctggcagtgg-3′). Following selection in puromycin (2 μg/ml), cells were lysed directly in 1 × Laemmli buffer (2% SDS, 10% glycerol, 5% 2-mercaptoethanol, 0.002% bromphenol blue and 0.0675 M Tris HCl, pH approx. 6.8) and resolved on SDS-PAGE gel of appropriate density. T26 antibody was used to detect NPMc+, while NPM1 was detected using a monoclonal antibody that recognizes specifically C-terminus of the protein (NPMc, [39]). Total β-catenin was detected using murine monoclonal antibody anti-β-catenin (Clone 14/β-catenin, BD). Active β-catenin was identified using a rabbit phospho-β-catenin (Ser552) (Cell Signaling). Such phosphorylation induces the accumulation of β-catenin in the nucleus where is binds to its target genes. AXIN2 was detected by a rabbit monoclonal antibody anti-Axin2 [EPR2005(2)]. An antibody anti-α-tubulin (Sigma-Aldrich) was used as a loading control. Images of blots were acquired using the ChemiDoc™ MP system (Bio-Rad) and analyzed/quantified using the built-in software.

### Patients' samples, immunofluorescence, qPCR and common mutation screening

For immunofluorescence experiments, bone marrow smears from 40 patients at diagnosis were obtained from the Hematology Department of the Tor Vergata University (Rome, Italy). Bone marrow samples were taken upon patients' informed consent and the Internal Review Board approved this research. Immunofluorescence staining was performed on smears fixed in 4% paraformaldehyde and permeabilized with 0.1% Triton X 100/0.2% BSA/PBS, as previously described [38]. The following antibodies were used: polyclonal anti-total β–catenin (Sigma) diluted 1:2000, anti-active β–catenin (ABC, Millipore) diluted 1:200 alone or together with a polyclonal antibody anti-NPMc+ (custom-made by Eurogentech) diluted 1:1000. Slides were analyzed with Olympus BX61 fluorescent microscope equipped with CoolSNAP EZ camera (Photometrics, Tucson, USA). Single colour images were taken with the Metapmorph software; overlay of different fluorescence channels and pseudocolor assignment was performed with ImageJ (Wayne Rasband, NIH, USA).

*AXIN2* expression (forward primer: 5′-ACAACAG CATTGTCTCCAAGCAGC-3′, reverse primer: 5′-GCGC CTGGTCAAACATGATGGAAT-3′) was analyzed in RNA from blasts of 30 AML patients bearing *NPM1* mutations and 10 AML patients with normal karyotype and no *NPM1* mutations using SybrGreen technology (for genetic characteristics see Supplementary Table S1) with TBP as a housekeeping control (forward primer: 5′-CGGCTG TTTAACTTCGCCTTC-3′, reverse primer: 5′-CACACGC CAAGAAACAGTGA-3′). Normal CD34+ progenitors, granulocytes and monocytes were included as control.

RNA samples from all patients were screened for the common AML mutations at known hotspots by direct Sanger sequencing of PCR products. The primers used for the screening are given in Supplementary Table S3.

## SUPPLEMENTARY MATERIALS


