# Supplementary material for: Nucleophosmin leukemogenic mutant activates Wnt signaling during zebrafish development
Source: Oncotarget. 2016 Jul 28;7(34):55302–12. doi: 10.18632/oncotarget.10878 (PMC5342418; doi:10.18632/oncotarget.10878)
Supplement: Supplementary file 1 [file oncotarget-07-55302-s001.pdf]

Nucleophosmin leukemogenic mutant activates Wnt signaling during zebrafish development

Supplementary Materials

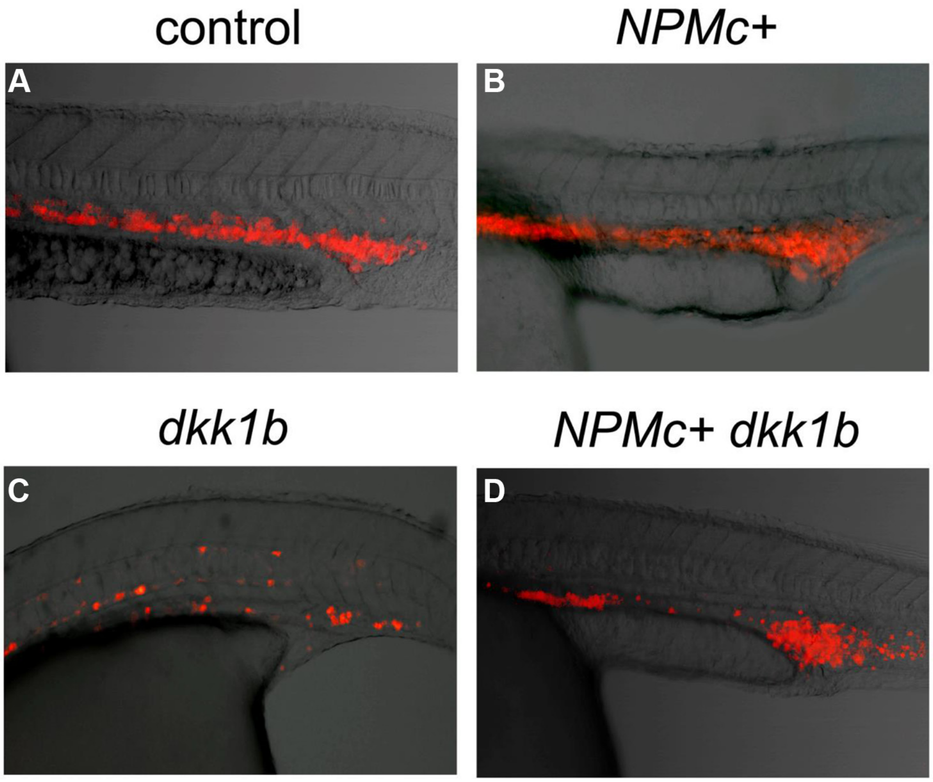

Supplementary Figure S1: Expression of the erythrocytic marker gata1 in 24 hpf embryos. *gata1* expression in *gata1*:*dsRED* embryos at 24 hpf, lateral view. (**A**) control embryo (33/33). Embryos were injected with *NPMc*+ mRNA (**B**; 28/31), *dkk1b* mRNA (**C**; 36/37) or co-injected with *NPMc*+ and *dkk1b* mRNAs (**D**; 8/35). Embryos were viewed with a Leica SP2 TCS confocal microscope.

Supplementary Table S1: Mutational status of the cohort shown in Figure 7

	FLT3	IDH1 R132	IDH2 R140	IDH2 R170	DNMT3a R882	N-RAS	K-RAS
**PT1**	*wt*	*wt*	*wt*	*wt*	*wt*	*wt*	*wt*
**PT2**	**ITD**	*wt*	**R140Q**	*wt*	*wt*	*wt*	*wt*
**PT3**	*wt*	*wt*	*wt*	*wt*	*wt*	*wt*	*wt*
**PT4**	*wt*	*wt*	*wt*	*wt*	**R882H**	*wt*	*wt*
**PT5**	*wt*	*wt*	*wt*	*wt*	*wt*	*wt*	*wt*
**PT6**	**ITD**	*wt*	*wt*	*wt*	**R882H**	*wt*	*wt*
**PT7**	*wt*	*wt*	*wt*	*wt*	*wt*	*wt*	*wt*
**PT8**	*wt*	*wt*	**R140Q**	*wt*	*NA*	*wt*	*wt*
**PT9**	**ITD**	*wt*	*wt*	*wt*	**R882H**	*wt*	*wt*
**PT10**	**ITD**	*wt*	*wt*	*wt*	*wt*	*wt*	*wt*
**PT11**	*wt*	*wt*	*wt*	*wt*	**R882C**	*wt*	*wt*
**PT12**	*wt*	*wt*	*wt*	*wt*	*wt*	*wt*	*wt*
**PT13**	*wt*	*wt*	*wt*	*wt*	**R882H**	*wt*	*wt*
**PT14**	*wt*	*wt*	*wt*	*wt*	*wt*	*wt*	*wt*
**PT15**	**ITD**	*wt*	*wt*	*wt*	*wt*	*wt*	*wt*
**PT16**	**D835**	*wt*	*wt*	*wt*	**R882H**	*wt*	*wt*
**PT17**	*wt*	*wt*	*wt*	*wt*	*wt*	*wt*	*wt*
**PT18**	**D835**	*wt*	*wt*	*wt*	**R882C**	*wt*	*wt*
**PT19**	**ITD**	*wt*	*wt*	*wt*	**R882C**	*wt*	*wt*
**PT20**	*wt*	*wt*	*wt*	*wt*	**R882C**	*wt*	*wt*
**PT21**	*wt*	*wt*	*wt*	*wt*	*wt*	*wt*	*wt*
**PT22**	*wt*	*wt*	**R140Q**	*wt*	*wt*	*wt*	*wt*
**PT23**	**ITD**	*wt*	*wt*	*NA*	*wt*	*wt*	*wt*
**PT24**	*wt*	*wt*	*wt*	*wt*	*wt*	*wt*	*wt*
**PT25**	*wt*	*wt*	*wt*	*wt*	**R882C**	*wt*	*wt*
**PT26**	**D835**	**R132H**	*wt*	*wt*	**R882C**	*wt*	*wt*
**PT27**	*wt*	*wt*	*wt*	*wt*	*wt*	*NA*	*wt*
**PT28**	*wt*	*wt*	*wt*	*wt*	*wt*	*wt*	*wt*
**PT29**	*wt*	*wt*	**R140Q**	*wt*	**R882H**	*NA*	*wt*
**PT30**	*wt*	*wt*	*wt*	*wt*	**R882C**	*wt*	*wt*
**PT31**	**ITD**	*wt*	*wt*	*wt*	*wt*	*wt*	*wt*
**PT32**	**ITD**	*wt*	*wt*	*wt*	*wt*	*wt*	*wt*
**PT33**	*wt*	*wt*	*wt*	**R172S**	*wt*	*NA*	*wt*
**PT34**	**ITD**	*wt*	*wt*	**R172K**	*wt*	*wt*	*wt*
**PT35**	*wt*	*wt*	**R140Q**	*wt*	**R882H**	*wt*	*wt*
**PT36**	*wt*	*wt*	*wt*	*wt*	*wt*	*wt*	*wt*
**PT37**	*wt*	*wt*	*wt*	*wt*	*wt*	*wt*	*wt*
**PT38**	*wt*	*wt*	*wt*	**R172K**	**R882H**	*wt*	*wt*
**PT39**	*wt*	*wt*	**R140Q**	*wt*	*wt*	*wt*	*wt*
**PT40**	*wt*	*wt*	*wt*	*wt*	*wt*	*wt*	*wt*

All patients had a normal karyoptype. Mutational status of *FLT3, IDH1, IDH2, DNMT3a, NRAS* and *KRAS* were investigated by Sanger sequencing of PCR products.
wt = wild type; NA = information not available.

Supplementary Table S2: Conditions for synthesis of mRNA probes used for in situ hybridization

AS Probe	Vector	Restriction Enzyme	RNA Polymerase
rx3	pBS-SK	XbaI	T7
pax2a	pGEM-3 zf(+/–)	BamHI	T7
gata2	pCRII-TOPO	BamHI	SP6
spi1	pBK-CMV	EcoRI	T7
tal1	pBK-CMV	SalI	T7
lmo2	pBK-CMV	EcoRI	T7
lcp1	pSPORt1	EcoRI	SP6

Supplementary Table S3: Primers used for screening for common AML mutations at reported hotspots

Primer:	5′-3′ sequence:
DNMT3a_F	GTGAGGACCATTACTACGAGGT
DNMT3a_R	TCCATGACCGGCCCAGCA
IDH1_132_F	CTTCAGAGAAGCCATTATCTG
IDH1_132_R	TCACTTGGTGTGTAGGTTATC
IDH2_140_F	GTTCAAGCTGAAGAAGATGTG
IDH2_140_R	TGAGATGGACTCGTCGGTG
IDH2_172_F	GAACTATCCGGAACATCCTG
IDH2_172_R	CTTGACACCACTGCCATC
K-RAS ex2+3_F	AGAGGCTCAGCGGCTCCCAG
K-RAS ex2+3_R	AGAAAGCCCTCCCCAGTCCTC
N-RAS ex2+3_F	CAACATTTTTCCCGGCTGTGGTC
N-RAS ex2+3_R	CGCTTAATCTGCTCCCTGTAGTG

IDH1/2 primer sequences were previously published in:
S. Abbas, S. Lugthart, F. G. Kavelaars, A. Schelen, J. E. Koenders, A. Zeilemaker, W. J. L. van Putten, A. W. Rijneveld, B. Löwenberg, P. J. M. Valk, D. Parsons, S. Jones, X. Zhang, F. Bleeker, S. Lamba, et al. Acquired mutations in the genes encoding IDH1 and IDH2 both are recurrent aberrations in acute myeloid leukemia: prevalence and prognostic value. Blood. 2010. 116(12): 2122–2126.
